# Evaluation of Screw-Retained Versus Screw-Cement-Retained Implant-Supported Fixed Prostheses for Marginal Bone Loss and Peri-Implant Soft Tissue Changes: An In Vivo Study

**DOI:** 10.7759/cureus.115513

**Published:** 2026-08-31

**Authors:** Pardeep K Bansal, Jaswinder Kaur, Harshdeep Bakshi, Shreya Kansal, Chhavi K, Preetika Bansal

**Affiliations:** 1 Prosthodontics, Dasmesh Institute of Research and Dental Sciences, Faridkot, IND; 2 Prosthodontics, Baba Farid University of Health Sciences, Faridkot, IND; 3 Dentistry, Dasmesh Institute of Research and Dental Sciences, Faridkot, IND

**Keywords:** bleeding on probing, cbct, dental implant, marginal bone loss, plaque index, screw-cement-retained prosthesis, screw-retained prosthesis

## Abstract

Aim: The influence of prosthetic retention design on early marginal bone loss and peri-implant soft tissue response remains clinically relevant. Screw-retained restorations provide retrievability and eliminate subgingival cement, whereas screw-cement-retained prostheses are designed to combine retrievability with the passive-fit and esthetic advantages of cementation.

Purpose: The purpose of this in vivo study was to compare screw-retained and screw-cement-retained implant-supported fixed prostheses in terms of marginal bone loss, plaque accumulation, and bleeding on probing over a six-month follow-up period.

Material and methods: Twenty partially edentulous participants restored with implant-supported fixed prostheses were included in this two-group analysis. Ten participants received screw-retained prostheses and 10 received screw-cement-retained prostheses. Marginal bone loss was assessed with cone beam computed tomography at the buccal, lingual, mesial, and distal aspects of each implant at three and six months after loading. Plaque index and bleeding on probing were recorded immediately after loading and at three and six months. Intragroup and intergroup comparisons were performed, and significance was set at P<0.05.

Results: Both groups showed a statistically significant increase in marginal bone loss from three to six months at all evaluated surfaces (P=0.001). At three months, screw-cement-retained prostheses showed significantly greater buccal bone loss than screw-retained prostheses (0.480 ± 0.181 mm versus 0.250 ± 0.190 mm; P=0.008). At six months, buccal bone loss remained significantly greater in the screw-cement-retained group (0.650 ± 0.205 mm) than in the screw-retained group (0.380 ± 0.169 mm; P=0.015). No significant differences were observed at the lingual, mesial, or distal surfaces. Plaque index scores were numerically higher in the screw-cement-retained group at three and six months, but the differences were not statistically significant. Bleeding on probing was comparable between groups.

Conclusions: Within the six-month follow-up period, screw-retained implant-supported fixed prostheses showed a more favorable buccal marginal bone response than screw-cement-retained prostheses. The screw-cement-retained design demonstrated acceptable peri-implant soft tissue outcomes, but it did not show a marginal bone loss advantage over the screw-retained design in this short-term evaluation.

## Introduction

Implant-supported fixed restorations have become a predictable treatment modality for the replacement of missing teeth [1,2]. Their long-term success depends not only on osseointegration but also on the preservation of peri-implant hard and soft tissues after prosthetic loading [3]. Marginal bone loss around implants is therefore an important radiographic parameter, while plaque index and bleeding on probing are useful clinical indicators of peri-implant soft tissue response [4-7]. The mode of prosthesis retention is selected during treatment planning and is influenced by available interocclusal space, implant angulation, occlusal access, esthetic demands, laboratory procedures, and retrievability [8,9]. Screw-retained (SR) prostheses have traditionally been favored when retrievability is important because they allow maintenance, repair, screw retightening, and evaluation of peri-implant tissues without damaging the prosthesis. In addition, they avoid the biological risk associated with retained subgingival cement. However, the occlusal access channel, screw loosening, and possible compromise in occlusal or esthetic design remain relevant limitations.

Screw-cement-retained prostheses were developed to combine the advantages of both screw and cement retention [10-12]. In this design, the crown may be cemented to the abutment extra orally or under direct control; excess cement can be removed before intraoral seating; and the final crown-abutment complex remains retrievable through the screw access. This design may reduce the risk of undetected cement remnants while retaining the possibility of prosthetic retrieval. Nevertheless, the effect of this hybrid retention design on early marginal bone remodeling and peri-implant soft tissue health requires further clinical evaluation. Previous studies comparing retention systems have reported inconsistent results [11,12]. Some studies have suggested a biologic advantage for screw-retained prostheses because cement remnants are eliminated, whereas others have suggested that cemented interfaces may compensate for minor misfit and distribute stress more favorably. The evidence concerning screw-cement-retained prostheses is still developing, particularly for short-term clinical changes assessed on multiple implant surfaces [12]. Therefore, the purpose of this in vivo study was to compare marginal bone loss, plaque index, and bleeding on probing around screw-retained and screw-cement-retained implant-supported fixed prostheses over a six-month period using radiographic and clinical parameters described in implant-retention literature. The null hypothesis was that no difference would be found between the two retention systems with respect to marginal bone loss or peri-implant soft tissue changes.

## Materials and methods

Ethical permission from the Institutional Ethics Committee has been obtained before the commencement of the study (IRB/24/4085). This two-group analysis was derived from an in vivo clinical investigation involving partially edentulous patients requiring implant-supported fixed prosthetic rehabilitation. The participants were selected randomly from the outpatient department after clinical and radiographic evaluation. Only the screw-retained and screw-cement-retained groups were considered for the present article. A total of 20 participants were included. Group SR comprised 10 participants restored with screw-retained implant-supported fixed prostheses. Group screw-cement-retained prosthesis (SCRP) comprised 10 participants restored with screw-cement-retained implant-supported fixed prostheses. Written informed consent was obtained before treatment.

Inclusion criteria

Adult participants who were 18 years or older, medically fit for implant surgery, partially edentulous, and had adequate bone width without the need for augmentation were included.

Exclusion criteria

Participants were required to have healthy oral tissues, no active infection or inflammation at the intended implant site, satisfactory oral hygiene, and compliance with the follow-up schedule. Patients with systemic illness that could compromise healing, poor oral hygiene, bleeding disorders, anticoagulant therapy, tobacco or alcohol dependence, immunocompromised status, history of chemotherapy or head and neck radiotherapy, psychiatric illness, or pregnancy were excluded.

Implant placement was performed under local anesthesia. A crestal incision was made, a full-thickness mucoperiosteal flap was reflected, and osteotomy preparation was performed with sequential drilling under continuous saline irrigation. The implant fixture (Figure 1a) was inserted according to the selected implant system protocol, a cover screw (Figure 1b) was placed, and the flap was sutured.

**Figure 1:**
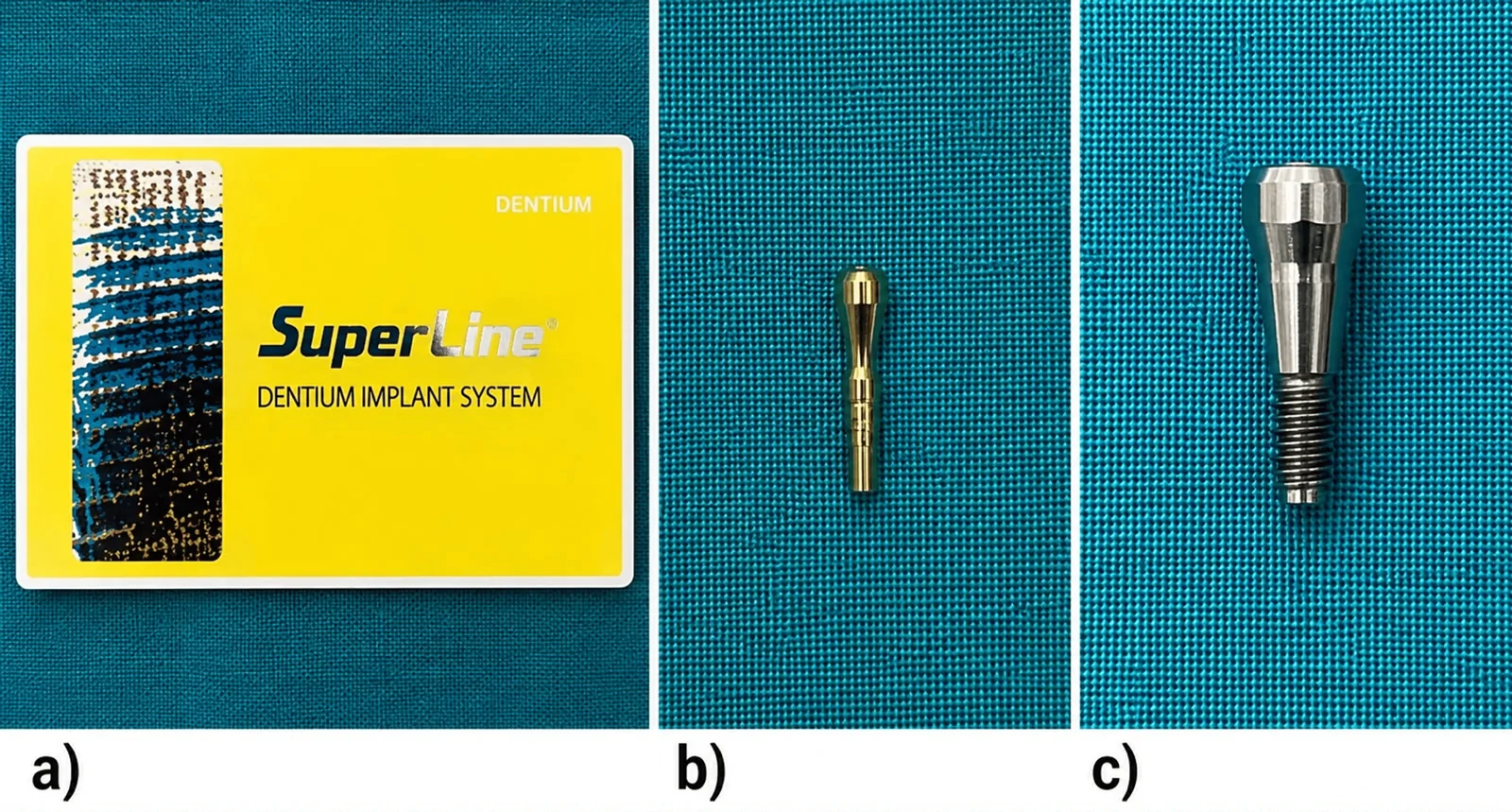
Implant system components (Dentium). (a) Superline/Dentium implant fixture, (b) cover screw, and (c) healing abutment.

A post-operative orthopantomogram (Figure 2) was obtained to verify implant position (mandibular posteriors). After a three-month osseointegration period, second-stage surgery was performed: the cover screw was replaced with a healing abutment (Figure 1c), and the prosthetic phase was initiated after soft-tissue healing.

**Figure 2:**
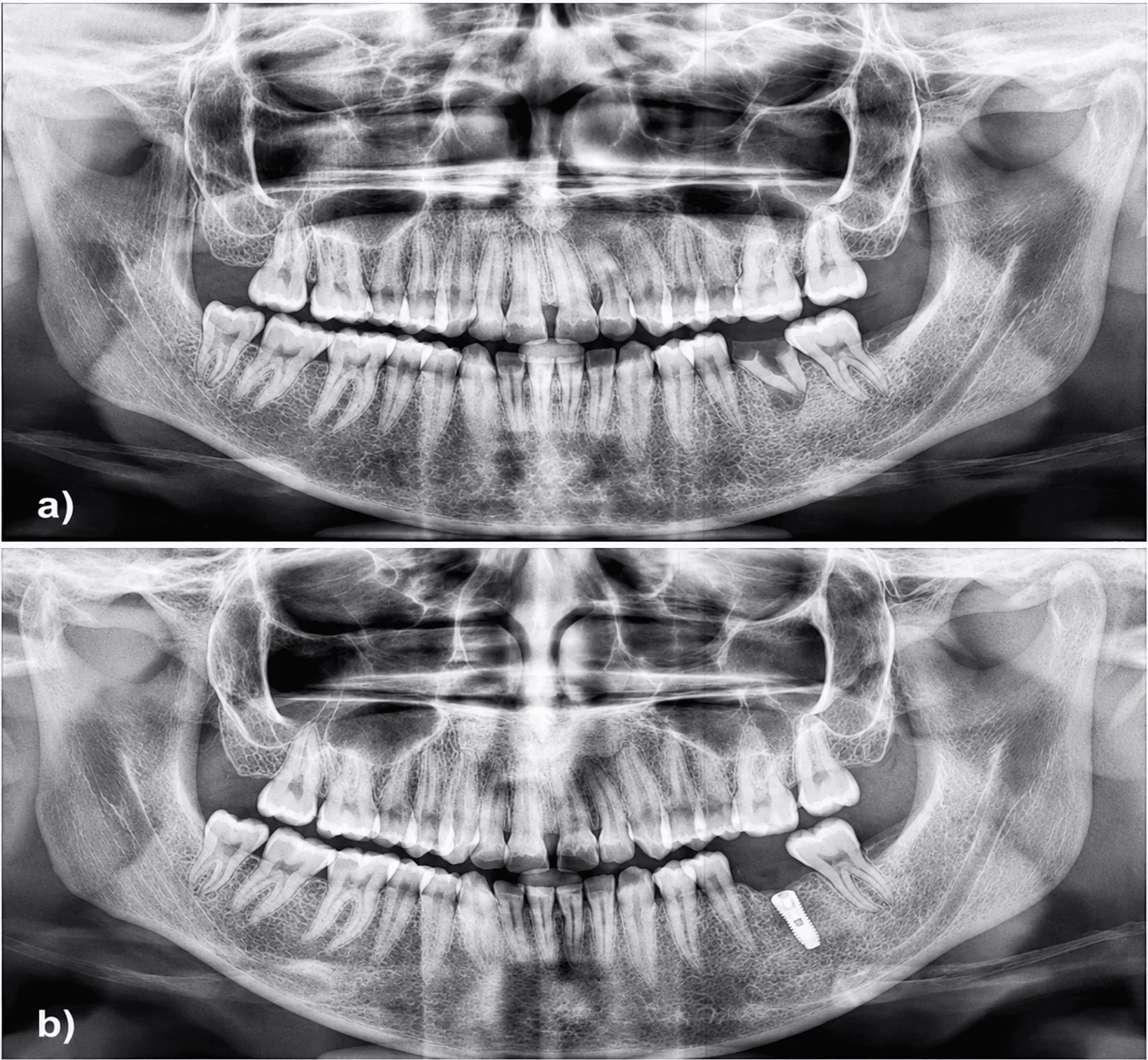
Orthopantomogram (OPG). (a) Pre-operative and (b) post-operative.

For the screw-retained group (Figures 3a, 3b), the prosthesis was fabricated with an occlusal access channel and was secured directly to the implant/abutment complex with a prosthetic screw. The screw-access channel was sealed after torqueing. For the screw-cement-retained group (Figures 3c, 3d), the crown component was cemented to the prepared abutment extraorally, excess cement was removed under direct visualization, and the crown-abutment complex was subsequently secured intraorally with a screw.

**Figure 3:**
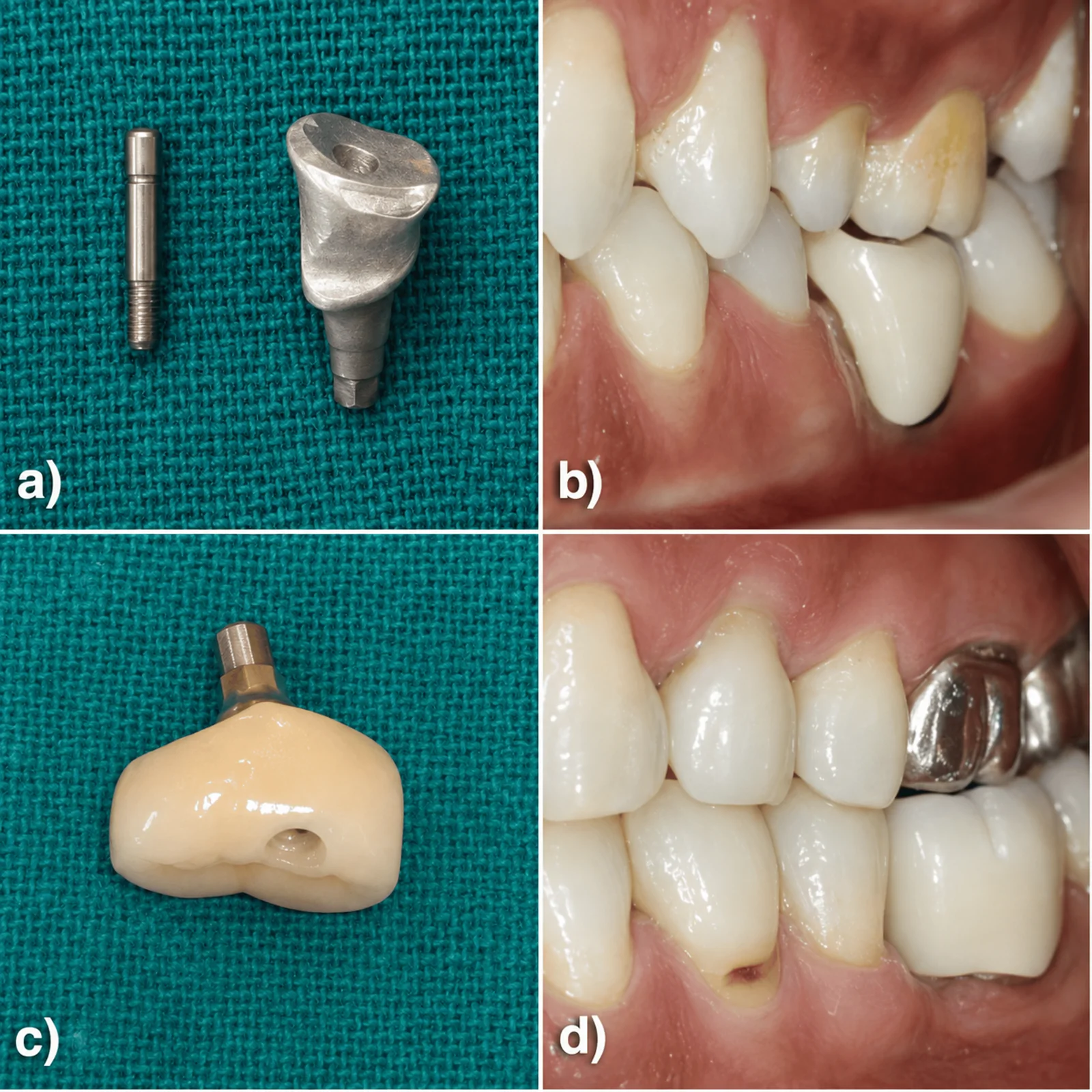
(a) Screw retained: metal trial framework with abutment screw, (b) intraoral placement of prosthesis, (c) SCRP: extraoral cementation of the crown to the prepared abutment, and (d) final SCRP cemented and secured intraorally. SCRP: screw-cement-retained prosthesis.

Marginal bone loss was evaluated by cone beam computed tomography (Figures 4, 5). Measurements were recorded at the buccal, lingual, mesial, and distal aspects of each implant at baseline, three, and six months after prosthetic loading. Plaque index and bleeding on probing (Figure 6) were recorded with a periodontal plastic probe immediately after loading and at three and six months. Bleeding on probing was recorded as present or absent.

**Figure 4:**
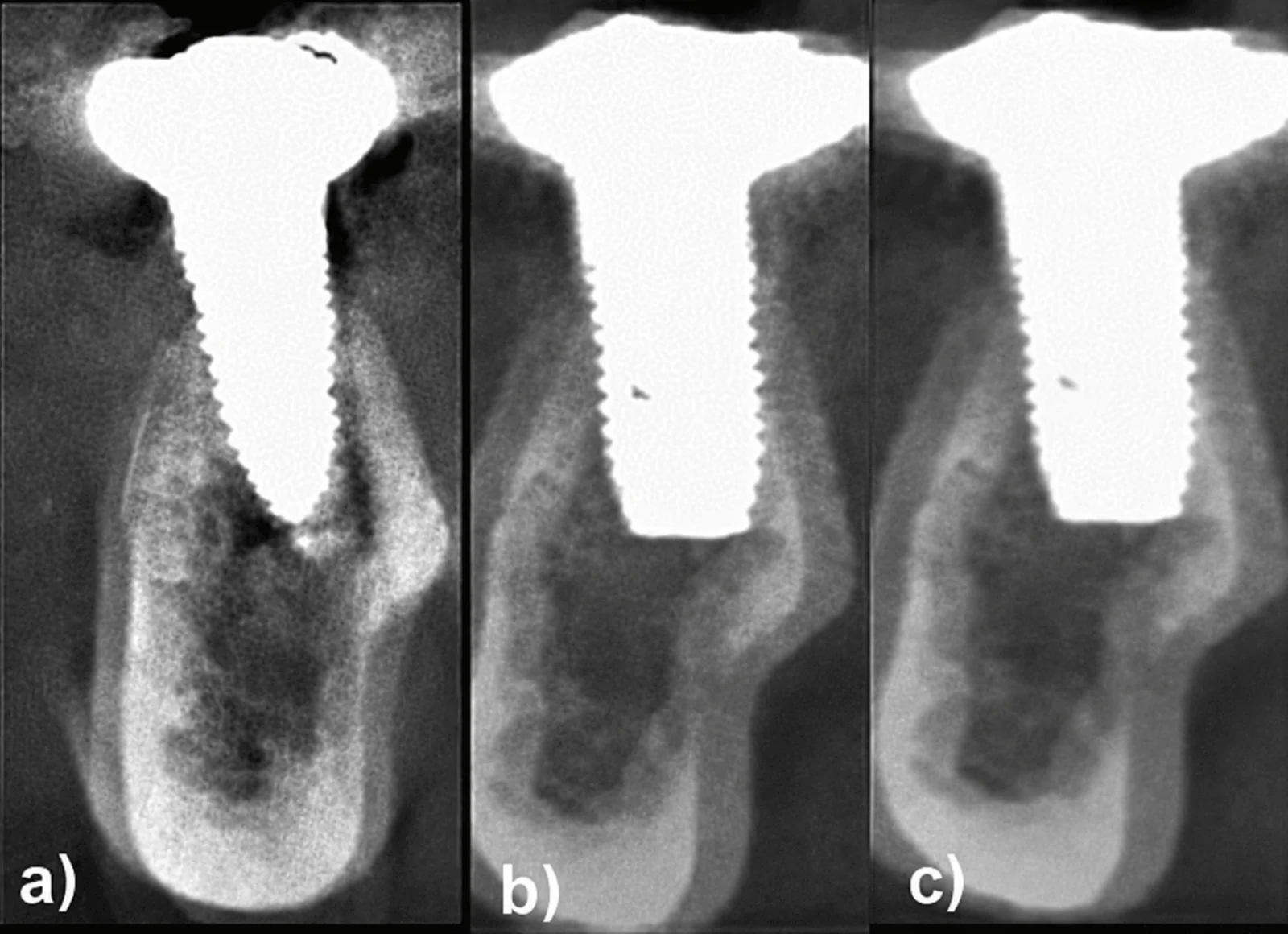
CBCT cross-sections of a representative screw-retained (SR) implant evaluating marginal bone levels. (a) Immediately after implant loading; (b) after three months of loading; and (c) after six months of loading. CBCT: cone-beam computed tomography.

**Figure 5:**
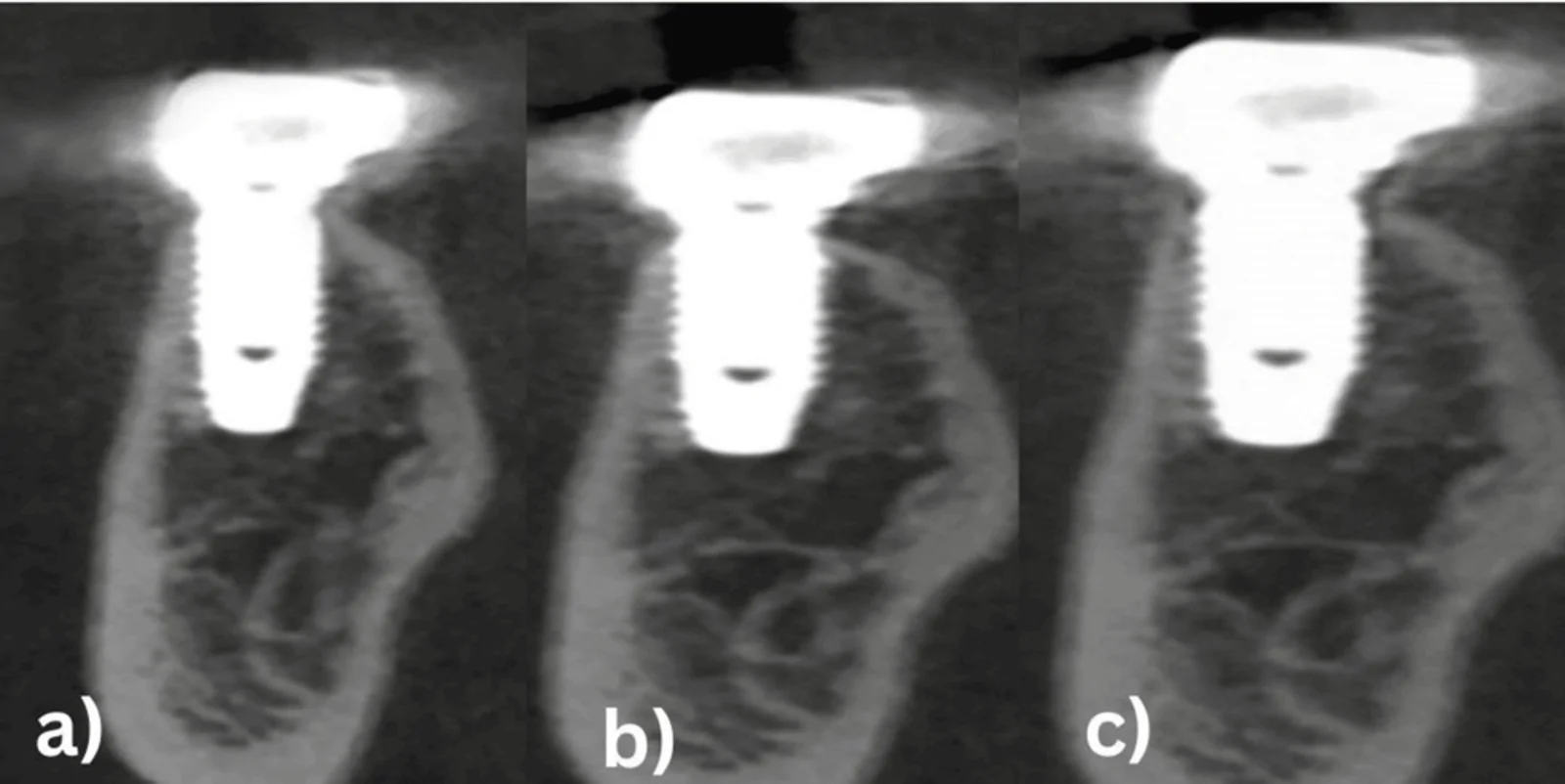
CBCT cross-sections of a representative SCRP implant evaluating marginal bone levels. (a) Immediately after implant loading; (b) after three months of loading; (c) after six months of loading. CBCT: cone-beam computed tomography; SCRP: screw-cement-retained prosthesis.

**Figure 6:**
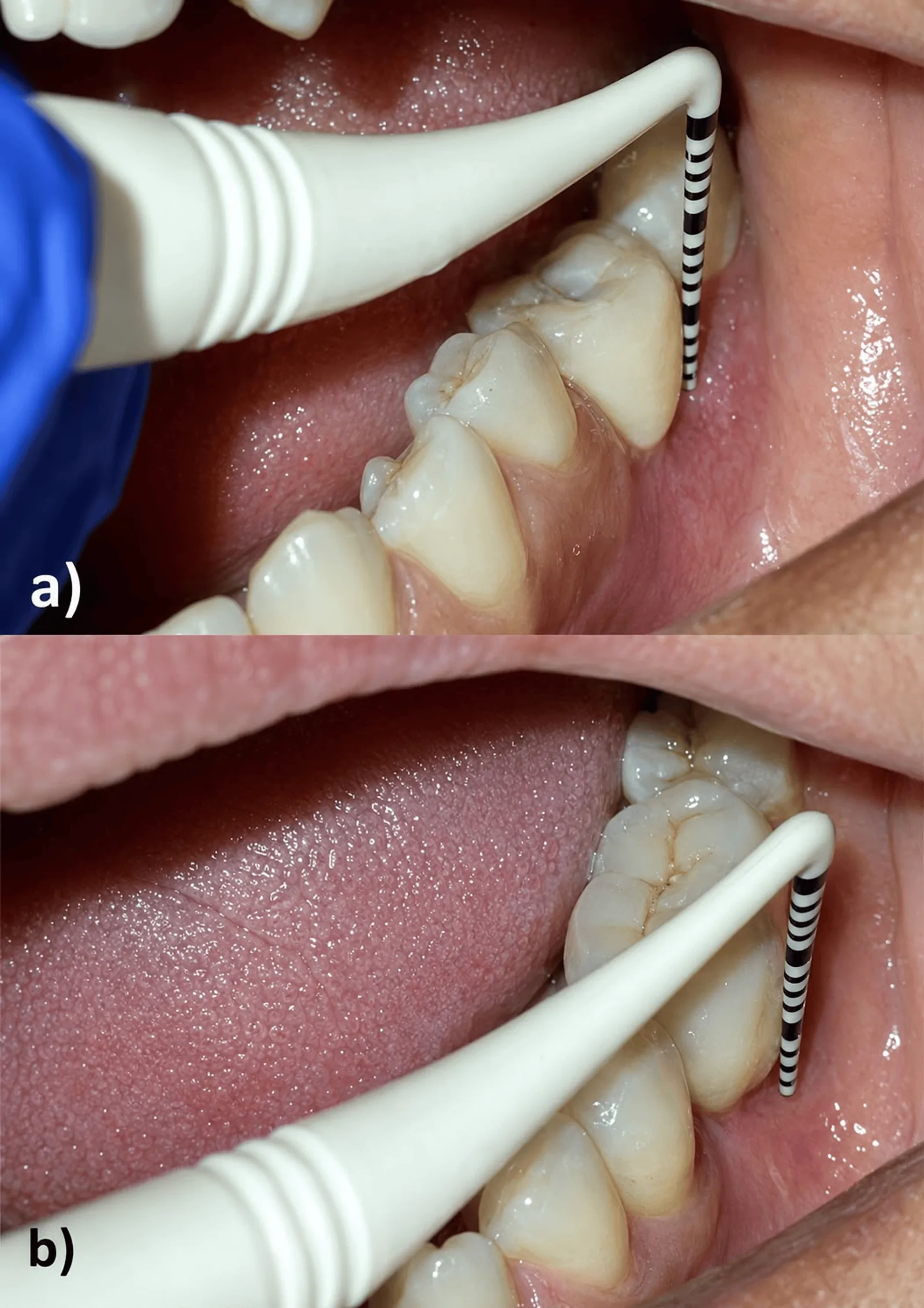
Soft-tissue evaluation utilizing a periodontal plastic probe. (a) Probing of the screw-retained prosthesis; (b) probing of the screw-cement-retained prosthesis.

Data were summarized as mean, standard deviation, standard error, and frequency where applicable. Intragroup comparisons were used to evaluate changes from three to six months. Intergroup comparisons between the screw-retained and screw-cement-retained groups were made for each surface and time interval. 

Statistical analysis

Results obtained were subjected to statistical analysis using the Statistical Package for the Social Sciences (SPSS) (version 26.0, IBM Corp., Armonk, NY, USA). Data were analyzed for normality using the Kolmogorov-Smirnov test and were found to be normally distributed. Therefore, a parametric significance test was applied. Descriptive statistics were performed. Data were presented as mean and standard deviation. Intergroup comparison was done using an independent t-test. Intragroup comparison was done using a paired t-test.

## Results

Twenty participants were evaluated, with 10 participants in each prosthetic retention group. Marginal bone loss increased from three to six months in both groups at all evaluated surfaces (Table 1).

**Table 1:** Summary of study groups, radiographic assessment schedule, measured implant surfaces, and clinical parameters. CBCT: cone-beam computed tomography; PI: plaque index; BOP: bleeding on probing.

Variable	Screw-retained	Screw-cement-retained
Participants (n)	10	10
Radiographic assessment	CBCT at baseline, three, and six months	CBCT at baseline, three, and six months
Measured implant surfaces	Buccal, lingual, mesial, distal	Buccal, lingual, mesial, distal
Clinical parameters	PI and BOP at baseline, three months, six months	PI and BOP at baseline, three months, six months

Marginal bone loss

At three months, screw-cement-retained prostheses showed significantly greater buccal bone loss than screw-retained prostheses. No statistically significant intergroup differences were observed on the lingual, mesial, or distal surfaces. At six months, the buccal surface again showed a significant difference favoring the screw-retained group, while the other surfaces remained statistically comparable (Table 2).

**Table 2:** Intergroup comparison of marginal bone loss between screw-retained and screw-cement-retained prostheses at three and six months. *Independent t-test was used. *P-value <0.05 was considered statistically significant.

Time	Surface	Screw-retained Mean ± SD (mm)	Screw-cement-retained prosthesis (SCRP) Mean ± SD (mm)	T-value	P-value
Three months	Buccal	0.250 ± 0.190	0.480 ± 0.181	2.982	0.008
Three months	Lingual	0.450 ± 0.246	0.370 ± 0.200	0.567	0.578
Three months	Mesial	0.330 ± 0.241	0.500 ± 0.194	1.265	0.222
Three months	Distal	0.200 ± 0.279	0.330 ± 0.170	1.095	0.288
Six months	Buccal	0.380 ± 0.169	0.650 ± 0.205	2.689	0.015
Six months	Lingual	0.660 ± 0.291	0.600 ± 0.205	0.329	0.746
Six months	Mesial	0.620 ± 0.262	0.740 ± 0.260	0.745	0.466
Six months	Distal	0.490 ± 0.367	0.520 ± 0.187	0.193	0.849

In the screw-retained group, bone loss increased significantly from three to six months at the buccal, lingual, mesial, and distal surfaces (P=0.001 for each surface). In the screw-cement-retained group, the increase from three to six months was also statistically significant at all surfaces (P=0.001).

Plaque index

Plaque index scores were 0 in both groups immediately after loading. At three and six months, the screw-cement-retained group showed higher mean plaque index scores than the screw-retained group; however, the intergroup differences were not statistically significant (Table 3).

**Table 3:** Intergroup comparison of plaque index scores at immediate, three-month, and six-month follow-up intervals. *Independent t-test was used. *P-value <0.05 was considered statistically significant.

Time interval	Screw-retained Mean ± SD	Screw-cement-retained prosthesis (SCRP) Mean ± SD	T-value	P-value
Immediately after loading	0.000 ± 0.000	0.000 ± 0.000	-	-
Three months	1.100 ± 0.530	1.475 ± 0.682	1.465	0.158
Six months	1.300 ± 0.563	1.675 ± 0.825	1.342	0.196

Bleeding on probing

Bleeding on probing was absent in all participants immediately after loading. At three months, bleeding was present in one participant in each group. At six months, bleeding was present in three participants in the screw-retained group and four participants in the screw-cement-retained group. The two groups were clinically comparable for bleeding on probing (Table 4).

**Table 4:** Distribution of bleeding on probing findings at immediate, three-month, and six-month follow-up intervals.

Time interval	Screw-retained absent/present	Screw-cement-retained prosthesis (SCRP) absent/present	Interpretation
Immediately after loading	10/0	10/0	Comparable
Three months	9/1	9/1	Comparable
Six months	7/3	6/4	Comparable

## Discussion

The present study compared early marginal bone loss and peri-implant soft tissue changes around screw-retained and screw-cement-retained implant-supported fixed prostheses. The null hypothesis was partly rejected because a significant difference was observed between the two groups for buccal marginal bone loss at both three and six months (Table 2). However, the two groups were statistically comparable on the lingual, mesial, and distal surfaces, and no significant differences were found in plaque index scores (Table 3). These findings should be interpreted in relation to previous reports showing that retention design can influence biological and technical outcomes around implant-supported restorations [13-17]. The screw-retained group demonstrated the most favorable buccal marginal bone response. At three months, buccal bone loss was 0.250 mm in the screw-retained group and 0.480 mm in the screw-cement-retained group. At six months, the corresponding values were 0.380 mm and 0.650 mm (Table 2). This difference may be clinically relevant because the buccal plate is often thin and may be more susceptible to early remodeling.

The absence of a cement interface in screw-retained restorations may also reduce the risk of plaque-retentive cement remnants at the peri-implant margin, a concern emphasized in previous reviews and clinical studies. Both groups showed a significant increase in marginal bone loss from three to six months (Table 2). This pattern is consistent with early post-loading remodeling and does not necessarily indicate implant failure when values remain within clinically acceptable ranges. The magnitude of bone loss in both groups remained within early clinical limits. The differences were mainly site-specific rather than generalized across all implant surfaces, suggesting that retention design may influence buccal remodeling more clearly than mesial, distal, or lingual bone behavior during short-term observation. Screw-cement-retained prostheses are intended to reduce the major biologic disadvantage of conventional cement-retained restorations by allowing extraoral cement removal and retrievability. In the present study, the screw-cement-retained group showed acceptable clinical behavior, but it did not demonstrate a marginal bone loss advantage over screw-retained prostheses (Table 2). This finding suggests that extraoral cement control may reduce but not completely eliminate the biologic and biomechanical factors associated with the hybrid design.

The plaque index findings showed numerically higher values in the screw-cement-retained group at both three and six months, although the differences were not statistically significant (Table 3). This descriptive trend may be related to the prosthetic contour, emergence profile, or the presence of a cemented crown-abutment interface, which have been discussed as factors affecting peri-implant hygiene and maintenance. The screw-retained group showed lower plaque scores throughout the follow-up period, which may reflect easier peri-implant maintenance and the absence of cement-related surface irregularities. Bleeding on probing was similar between the two groups (Table 4). Both groups showed an increase in bleeding frequency from baseline to six months, but the difference between them was small. This finding suggests that during a six-month follow-up period, both retention systems can maintain clinically acceptable soft-tissue health when patients are carefully selected, and oral hygiene instructions are reinforced. The findings of the present study should be interpreted with caution. The sample size was small, the follow-up period was limited to six months, and the clinical soft-tissue parameters were evaluated over a short observation interval. The original clinical investigation included three retention designs, whereas the present article focuses only on the screw-retained and screw-cement-retained groups. Longer-term randomized clinical studies with larger samples are required to determine whether the early buccal bone pattern observed in this study persists over time, as long-term and systematic evidence remains important for interpreting retention-system outcomes.

## Conclusions

Both screw-retained and screw-cement-retained implant-supported fixed prostheses showed a significant increase in marginal bone loss from three to six months. Screw-retained prostheses demonstrated significantly lower buccal marginal bone loss than screw-cement-retained prostheses at both three and six months. No significant intergroup differences were observed at the lingual, mesial, or distal surfaces. Plaque index values were numerically lower in the screw-retained group, but the difference between the groups was not statistically significant. Bleeding on probing was comparable between the two retention systems during the six-month follow-up. Within the limitations of this short-term study, screw-retained prostheses showed a more favorable early buccal peri-implant bone response, while screw-cement-retained prostheses remained a clinically acceptable retrievable alternative.
